# Exploring the causal associations between diet-derived circulating antioxidants and the risk of endometriosis: a Mendelian randomization study

**DOI:** 10.3389/fnut.2024.1453147

**Published:** 2024-09-09

**Authors:** Xiaoming Li, Zhen Xie, Hongbing Qiu, Xiaofeng Xie, Lusha Liu

**Affiliations:** ^1^Department of Gynecology, Xingtai People’s Hospital, Xingtai, China; ^2^Department of Gynecology, The Fourth Hospital of Hebei Medical University, Shijiazhuang, China

**Keywords:** endometriosis, oxidative stress, circulating antioxidants, Mendelian randomization, causality

## Abstract

**Background:**

Numerous observational studies and randomized controlled trials have recently revealed the associations between circulating antioxidants and the risk of endometriosis, while the underlying causal relationship remains unclear. This study aimed to investigate the causal association between genetically determined circulating antioxidants and the risk of endometriosis using Mendelian randomization (MR).

**Methods:**

A two-sample MR analysis was conducted using publicly available summary data from genome-wide association studies (GWAS) to investigate the causal impact of genetically determined absolute circulating antioxidants (such as ascorbate, retinol, β-carotene, and lycopene) and their metabolites (including α-and γ-tocopherol, ascorbate, and retinol) on the risk of endometriosis. The study used inverse variance weighted (IVW) or Wald ratio analyses as the primary estimation method and also conducted sensitivity analyses to assess heterogeneity and pleiotropy.

**Results:**

No significant causality was observed for genetically determined circulating antioxidants and the risk of endometriosis. The pooled odds ratios (ORs) for absolute circulating antioxidants were 0.62 (95% CI: 0.32–1.18, retinol), 0.95 (95% CI: 0.79–1.15, β-carotene), 1.01 (95% CI: 0.95–1.08, lycopene), and 1.00 (95% CI: 0.99–1.02, ascorbate, expressed as a Wald ratio). The pooled ORs indicating the EM risk per unit increase in circulating antioxidant metabolites were 1.04 (95% CI: 0.82–1.33, γ-tocopherol), 0.91 (95% CI: 0.57–1.46, α-tocopherol), 1.03 (95% CI: 0.99–1.07, retinol), and 0.96 (95% CI: 0.87–1.06, ascorbate).

**Conclusion:**

Our study demonstrated that increased levels of diet-derived circulating antioxidants were not significantly associated with a reduced risk of endometriosis.

## 1 Introduction

Endometriosis (EM) is characterized by endometrial-like tissues growing outside the uterine cavity, affecting approximately 10% of childbearing women globally (1). Although chronic pelvic pain and infertility are common symptoms of EM, due to the heterogeneity of these symptoms in the early stages of the disease, up to 65% of women are initially misdiagnosed (2). Treatments for EM, including hormonal and non-hormonal drugs and surgery, can only alleviate symptoms and not cure the disease (3). Various theories about the pathogenesis of EM, such as immunity, genetics, and environment, have been proposed but not fully elucidated (1). Currently, increasing evidence indicates that oxidative stress plays a significant role in the pathophysiological process of EM (4–7).

Existing studies suggest that oxidative stress markers are elevated in EM patients compared to those in controls (8, 9). Oxidative stress is characterized by an imbalance between reactive oxygen species (ROS) and antioxidants， which manifests an abundance of ROS and a deficiency of antioxidant mechanisms (10). Oxidative stress increases the levels of IL-10 in the serum and peritoneal fluid of patients with EM by enhancing the activity of the NF-κB signaling pathway to promote the development of EM (11, 12). Additionally, decreased activity of endogenous antioxidant enzymes, such as SOD or GPx, may also show a positive correlation with the severity of EM (13). Interestingly, several observational studies have demonstrated that EM patients have a lower intake of antioxidant vitamins A, C, and E (14, 15). Meanwhile, randomized clinical trials (RCTs) found that a high-antioxidant diet could reduce the levels of oxidative stress markers and improve disease symptoms (16–18). The common diet-derived antioxidants include vitamin A (retinol), vitamin E (α-and γ-tocopherol), vitamin C (ascorbate), and carotenoids (β-carotene, lycopene). Among them, in EM, vitamin C can effectively reduce free radicals and reactive oxygen species (ROS), thereby inhibiting the adhesion and growth of endometrial cells; vitamin A can suppress the secretion of IL-6 and VEGF, further inhibiting the inflammatory response and growth of ectopic lesions (19). These results indicate that a high-antioxidant diet or antioxidant supplements may represent a promising new approach for reducing the incidence of EM and improving treatment outcomes. However, it is important to note that previous observational studies were limited by small sample sizes and potential confounding factors, and most RCTs on antioxidants primarily focused on improving disease symptoms, thus leaving the causal relationship between antioxidants and the risk of endometriosis still unclear.

Mendelian randomization (MR) analysis uses genetic variables, which are determined at the time of fertilization, as instrumental variables to assess the causal relationship between exposures and outcomes. This approach minimizes the influence of confounding factors and reverse causality offering a reliable estimate of the causal association between exposure and outcomes under specific assumptions (20). Therefore, in this study, we conducted a two-sample MR analysis to evaluate the causal relationship between genetically determined diet-derived circulating antioxidants and EM.

## 2 Materials and methods

### 2.1 Study design

This two-sample MR analysis based on summary statistics from genome-wide association studies (GWASs) was conducted to investigate the causal relationship between diet-derived circulating antioxidants and the risk of EM. We used the two antioxidants’ phenotypes as exposure: absolute circulating antioxidants and circulating antioxidant metabolites. Among them, absolute circulating antioxidants contain lycopene, retinol, β-carotene, and ascorbate, and circulating antioxidant metabolites include retinol, ascorbate, α-tocopherol, and γ-tocopherol. Furthermore, the selected instrumental variables (IVs) should meet three key assumptions: first, the IVs must be related to the exposure factor; second, IVs are unrelated to confounders influencing exposure and outcome; and third, IVs affect the outcome variable only through the exposure factor (21). The IVs of circulating antioxidants are shown in Supplementary Table S1.

### 2.2 Genetic instrumental variables for antioxidants

For IVs of absolute circulating antioxidants, five single-nucleotide polymorphisms (SNPs) [*p* < 5×10-6, linkage disequilibrium (LD) < 0.001] linked with lycopene were identified from a GWAS study involving 441 Amish adults (22); 3 SNPs (*p* < 5 × 10–8, LD < 0.2) associated with β-carotene were identified from a GWAS of Nurses’ Health study with 2,344 participants (23); and 2 SNPs (*p* < 5 × 10–8, LD < 0.001) associated with retinol were derived from a GWAS involving 5,006 Caucasian individuals (24). We obtained summary data for one SNP for ascorbate from a meta-analysis involving five studies with >15,000 individuals (*p* < 2 × 10–7) (25).

For circulating antioxidant metabolites, IVs of genetically determined retinol, ascorbate, α-tocopherol, and γ-tocopherol were obtained from two published GWAS studies of the European population (*p* < 1 × 10–5) (24, 26). In total, we identified 24 SNPs for retinol (participants = 1957), 11 SNPs for α-tocopherol (participants = 7,276), 13 SNPs for γ-tocopherol (participants = 5,822), and 14 SNPs for ascorbate (participants = 2063), which were all derived from European population studies. When LD was greater than 0.001, we selected the IVs with the smallest *p*-value. The proportion of variability (R2) and F-statistic were used to evaluate the strength of these IVs (27). The F-statistic >10 (formula: F = beta2 /SE2) for each SNP was recommended for subsequent MR analysis to ensure the robust association between IVs and exposure factors. The *R*^2^ value for each SNP was determined using the formula *R*^2^ = (β× $\sqrt{2xMAF\left(1-MAF\right)}$ ^2^) (MAF: the minor allele frequency; β: the effect of the SNP on the endometriosis.) or retrieved from the original study. Moreover, based on the PhenoScanner database, the potential SNPs associated with confounders were removed (28). The information on SNPs related to circulating antioxidants is shown in Supplementary Table S2.

### 2.3 Genetic instrumental variables for endometriosis

GWAS summary statistics for EM were derived from the OpenGWAS database (29), and the GWAS ID is Finn-b-N14_ Endometriosis, which includes 8,288 cases and 68,969 controls of European participants. Genetic variants at the genome-wide significance level *p* < 5 × 10–6 were selected as IVs for EM. If appropriate proxy SNPs were not available for SNPs missing in the outcome GWAS, those SNPs were subsequently excluded from the analysis.

The ethical approval of all these studies had been acquired by related review committees in their respective institutions. In this study, no new data were collected, and no new ethical approval was needed.

### 2.4 Statistical analysis

MR was conducted using the “TwoSampleMR” and “MRPRESSO” packages (R.4.1.2 version). We mainly used the inverse-variance weighted (IVW) method to assess the causal relationship between circulating antioxidants and EM, while MR-Egger, simple mode, weighted median, and weighted mode were used to further verify the results. When only one SNP was used as an IV for exposure, the Wald ratio was used to conduct MR analysis. MR-Egger intercept and MR-PRESSO were used to assess pleiotropy (30), and Cochran’s Q test was used to identify heterogeneity (31). If the *p*-value was greater than 0.05, indicating no evidence of heterogeneity, a random-effects model was adopted; otherwise, a fixed-effects model was used for the analysis. Additionally, we conducted a leave-one-out sensitivity analysis to further evaluate the robustness of our results.

## 3 Results

### 3.1 Selection of genetic instrumental variables

The relative summary information of IVs involved in EM and circulating antioxidants is shown in Supplementary Tables S3, S4. In MR analysis, a total of 14 SNPs for ascorbate, 23 SNPs for retinol, 8 SNPs for α-tocopherol, and 10 SNPs for γ-tocopherol in circulating antioxidant metabolites, along with 1 SNP for ascorbate, 2 SNPs for retinol, 2 SNPs for β-carotene, and 5 SNPs for lycopene in absolute circulating antioxidants, were included.

### 3.2 Effect of circulating antioxidants on the risk of endometriosis

**Figure 1:**
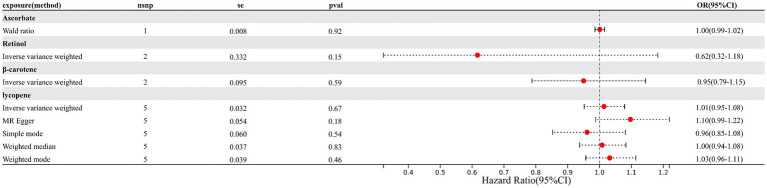
Causal association between absolute circulating antioxidants and endometriosis.

**Figure 2:**
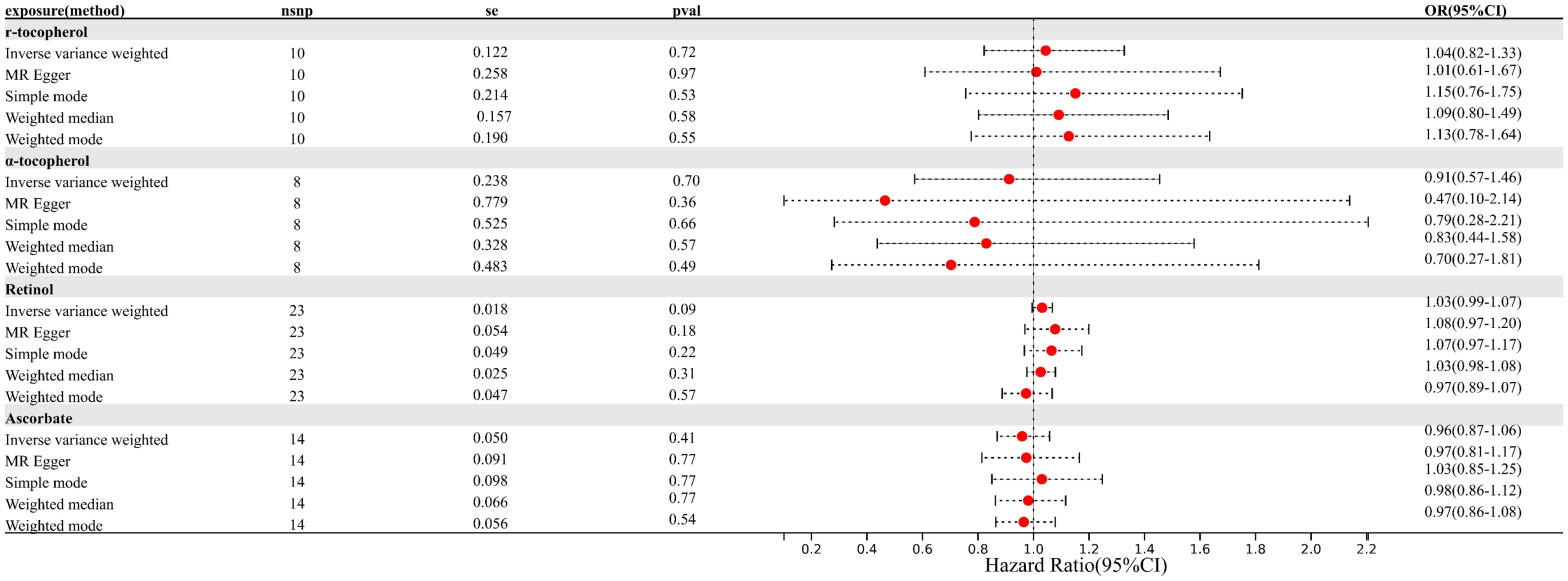
Causal association between circulating antioxidant metabolites and endometriosis.

As depicted in Figures 1, 2 (forest plot), there were no significant causal relationships observed between elevated levels of antioxidants and the risk of EM, consistently for both absolute circulating antioxidants and circulating antioxidant metabolites. The results of IVW suggested that ORs for absolute circulating antioxidants were 0.62 (95% CI: 0.32–1.18, *p* = 0.15, retinol), 0.95 (95% CI: 0.79–1.15, *p* = 0.59, β-carotene), 1.01 (95% CI: 0.95–1.08, *p* = 0.67, lycopene), and 1.00 (95% CI: 0.99–1.02, *p* = 0.92, ascorbate, expressed as Wald ratio), and ORs of circulating antioxidant metabolites were 1.04 (95% CI: 0.82–1.33, *p* = 0.72, γ-tocopherol), 0.91 (95% CI: 0.57–1.46, *p* = 0.70, α-tocopherol), 1.03 (95% CI: 0.99–1.07, *p* = 0.09, retinol), and 0.96 (95% CI: 0.87–1.06, *p* = 0.41, ascorbate). Due to the negative results, we did not further evaluate horizontal pleiotropy and heterogeneity. In conclusion, these pieces of evidence suggest that there are no significant causal associations between increased levels of diet-derived circulating antioxidants and a reduced risk of EM.

## 4 Discussion

In this study, our results did not reveal a genetic causal relationship between diet-derived circulating antioxidants and the risk of EM based on MR analyses, as evidenced by the consistent findings obtained from both the absolute levels of circulating antioxidants and the levels of antioxidant metabolites in the body.

Oxidative stress is a hallmark of EM, with patients often exhibiting increased oxidative stress markers and decreased antioxidant capacity. Antioxidants, including vitamins C, E, and A, play a pivotal role in modulating the progression of EM by regulating the balance between ROS production and antioxidant defense mechanisms (32). Previous observational studies have demonstrated a negative association between dietary antioxidant intake and the risk of EM. For instance, a study conducted by Mier et al. used a food frequency questionnaire to compare antioxidant intake in women with and without EM, and it revealed that EM patients had a lower intake of vitamins A, C, and E (14). Another prospective cohort study suggested that a higher intake of fruits, particularly citrus fruits, is associated with a lower risk of EM, as citrus fruits are rich in vitamins A and C (19). In addition, clinical trials have shown that supplementation with vitamins C and E in EM patients can effectively reduce the levels of inflammatory cytokines and oxidative stress markers, leading to an improvement in clinical symptoms (14, 33). A randomized triple-blind clinical trial conducted by Amini et al. demonstrated that treatment with vitamins C and E could effectively reduce the level of oxidative stress markers and alleviate symptoms such as pelvic pain, dysmenorrhea, and dyspareunia (16). A previous study has shown that vitamin E supplementation could reduce EM-related pelvic pain (18). However, there remains a scarcity of RCTs investigating whether antioxidant supplementation can effectively reduce the risk of EM. A previous MR study on circulating antioxidants has shown that the impact of genetic variants on antioxidant levels is generally comparable to that achieved through dietary supplementation (34). Our MR study is the first to indicate that there is no causal association between circulating antioxidants and the risk of EM, suggesting that dietary supplements increasing blood antioxidant levels may not lower the risk of EM in healthy adults with sufficient nutrition. It is worth noting that our results do not contradict the hypothesis that oxidative stress plays a significant role in the pathogenesis of EM. This finding could be explained by the fact that circulating antioxidant levels do not necessarily correspond to antioxidant nutritional intake (35). Therefore, despite the absence of a causal association between diet-derived circulating antioxidants and the risk of EM, patients may still benefit from antioxidant supplementation to reduce damage due to EM.

There are two major strengths in this study. First, we evaluated the causal relationship between EM and two different sources of antioxidants: absolute circulating antioxidants and their metabolites. The consistency of the two results strongly supports our conclusion. Second, to fully explore the causal relationship between exposure and outcome, we not only used the IVW method but also further validated our findings using additional methods such as MR-Egger and the weighted median approach. However, this study also has some limitations that need to be noted. First, the GWAS summary data for EM were derived from European populations, and the applicability of the findings to other populations remains uncertain. Second, when the number of SNPs is less than three, MR analysis only uses the Wald ratio or IVW, while other methods such as MR-Egger and weighted median cannot be used for assessing the causal relationship. Thus, we selected absolute circulating antioxidants and antioxidant metabolites to assess the antioxidant content of the body in order to enhance the reliability of our results.

## 5 Conclusion

In summary, our MR results have not found a genetic association between circulating antioxidants and the risk of EM. This study suggests that for healthy adults without nutritional deficiencies, it is not recommended to take additional antioxidants to prevent EM. In the future, further validation of our current findings through large-scale GWASs is still needed.

## Data Availability

The original contributions presented in the study are included in the article/Supplementary material, further inquiries can be directed to the corresponding author.
