# Supplementary material for: Exploring the causal associations between diet-derived circulating antioxidants and the risk of endometriosis: a Mendelian randomization study
Source: Front Nutr. 2024 Sep 9;11:1453147. doi: 10.3389/fnut.2024.1453147 (PMC11416951; doi:10.3389/fnut.2024.1453147)
Supplement: Supplementary file 1 [file Data_Sheet_1.docx]

Supplementary Material

# Supplementary Figures and Tables

## Supplementary Figures


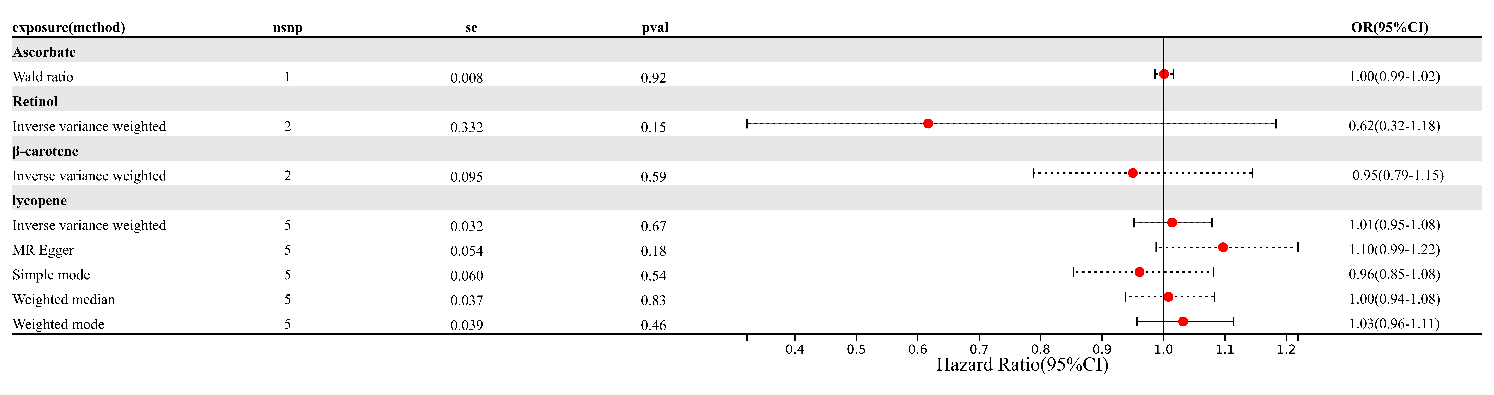


Fig 1. Causal association between absolute circulating antioxidants with endometriosis.


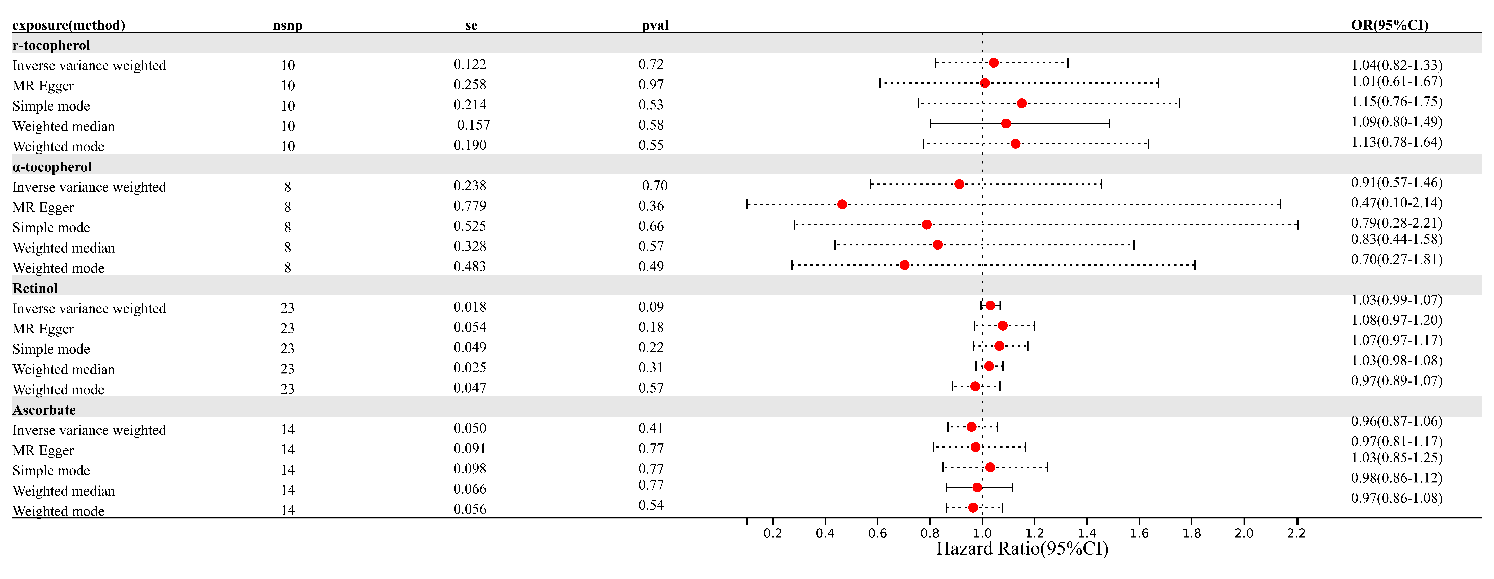


Fig 2. Causal association between circulating antioxidants metabolites with endometriosis.

## Supplementary Tables

**Supplementary Table S1. Instrumental variables of circulating dietary-derived antioxidants.**

**
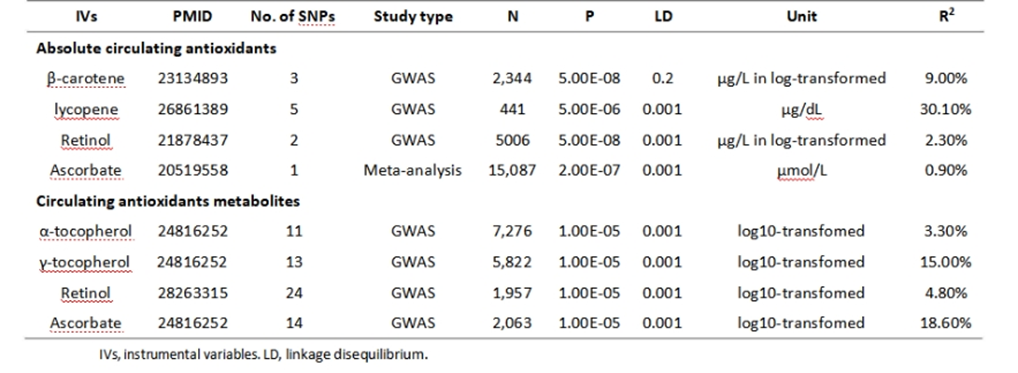
**

**Supplementary Table S2. SNPs of circulating antioxidants in the Mendelian randomization analysis**

**
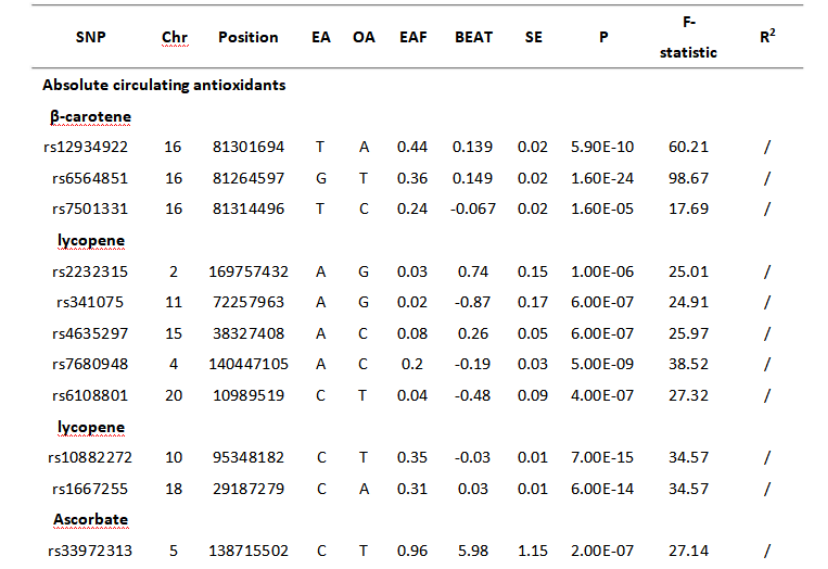
**

**
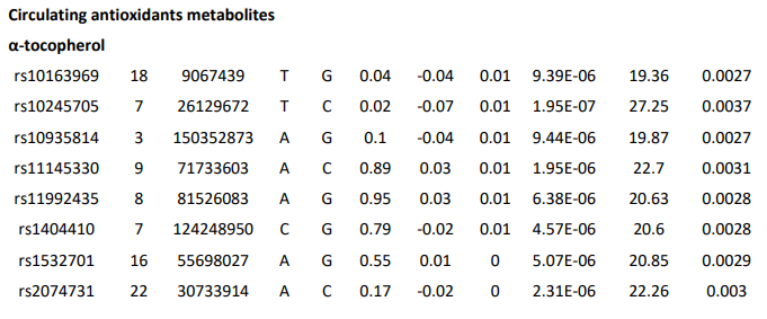
**

**
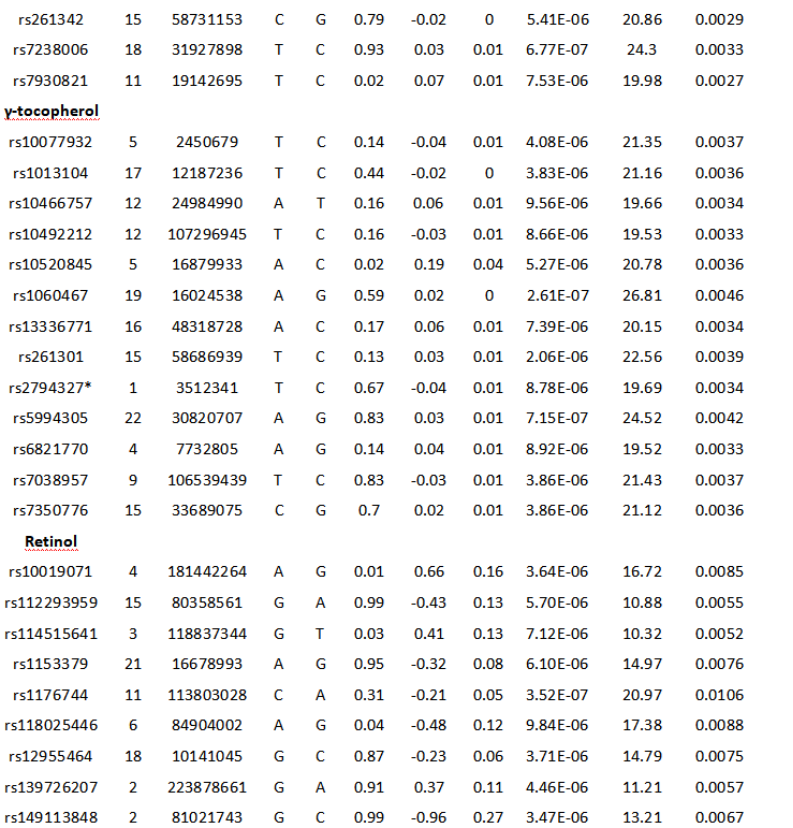
**

**
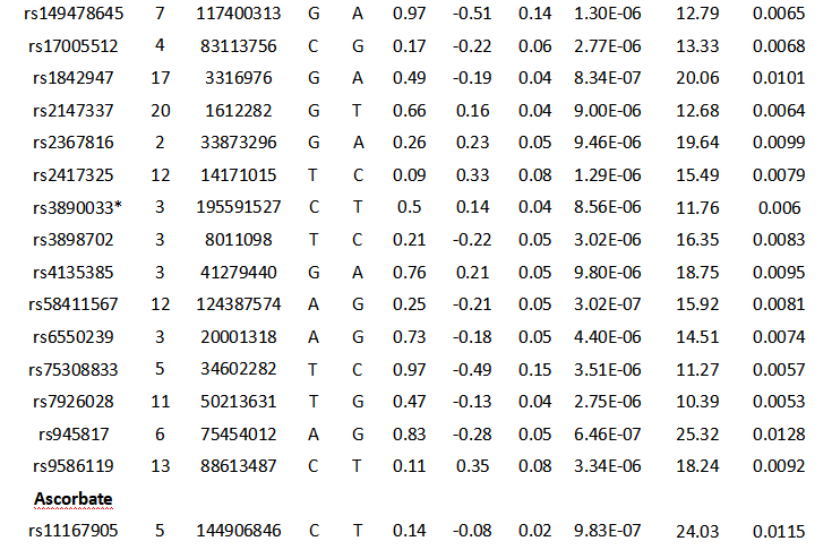
**

**
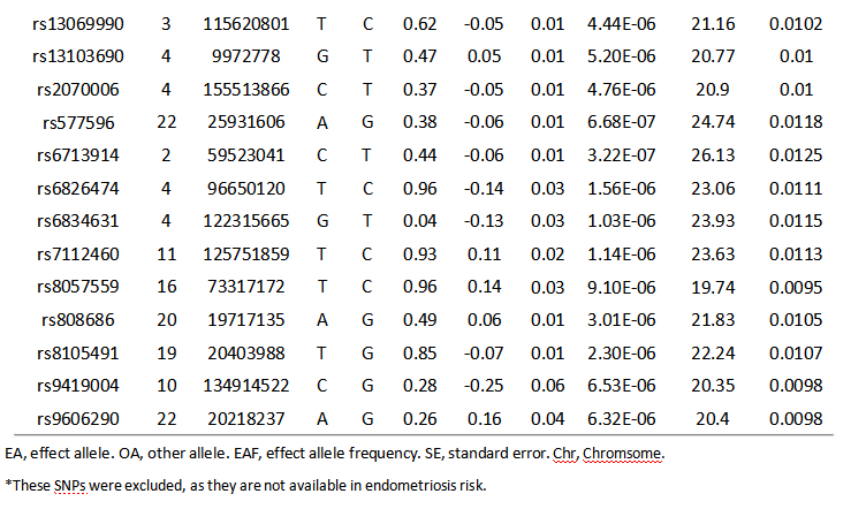
**

**Supplementary Table S3. The results of mendelian randomization between circulating antioxidants and endometriosis**

| **antioxidants** | **nSNP** | **method** | **Beta** | **SE** | **P-value** | **OR (95%CI)** |
| --- | --- | --- | --- | --- | --- | --- |
| β-carotene | 2 | IVW | -0.051 | 0.095 | 0.59 | 0.95 (0.79-1.15) |
| lycopene | 5 | IVW | 0.014 | 0.032 | 0.67 | 1.01 (0.95-1.08) |
|  | 5 | MR Egger | 0.093 | 0.054 | 0.18 | 1.10 (0.99-1.22) |
|  | 5 | Simple mode | -0.040 | 0.060 | 0.54 | 0.96 (0.85-1.08) |
|  | 5 | Weighted median | 0.008 | 0.037 | 0.83 | 1.00 (0.94-1.08) |
|  | 5 | Weighted mode | 0.032 | 0.039 | 0.46 | 1.03 (0.96-1.11) |
| Retinol | 2 | IVW | -0.483 | 0.332 | 0.15 | 0.62 (0.32-1.18) |
| Ascorbate | 1 | Wald ratio | 0.001 | 0.008 | 0.92 | 1.00 (0.99-1.02) |

IVW: inverse variance weighted.

**Supplementary Table S4. The results of mendelian randomization between circulating antioxidants metabolites and endometriosis**

| **antioxidants** | **nSNP** | **method** | **Beta** | **SE** | **P-value** | **OR (95%CI)** |
| --- | --- | --- | --- | --- | --- | --- |
| r-tocopherol | 10 | IVW | 0.043 | 0.122 | 0.72 | 1.04 (0.82-1.33) |
|  | 10 | MR Egger | 0.010 | 0.258 | 0.97 | 1.01 (0.61-1.67) |
|  | 10 | Simple mode | 0.141 | 0.214 | 0.53 | 1.15 (0.76-1.75) |
|  | 10 | Weighted median | 0.087 | 0.157 | 0.58 | 1.09 (0.80-1.49) |
|  | 10 | Weighted mode | 0.119 | 0.19 | 0.55 | 1.13 (0.78-1.64) |
| α-tocopherol | 8 | IVW | -0.092 | 0.238 | 0.70 | 0.91 (0.57-1.46) |
|  | 8 | MR Egger | -0.766 | 0.779 | 0.36 | 0.47 (0.10-2.14) |
|  | 8 | Simple mode | -0.238 | 0.525 | 0.66 | 0.79 (0.28-2.21) |
|  | 8 | Weighted median | -0.186 | 0.328 | 0.57 | 0.83 (0.44-1.58) |
|  | 8 | Weighted mode | -0.352 | 0.483 | 0.49 | 0.70 (0.27-1.81) |
| Retinol | 23 | IVW | 0.031 | 0.018 | 0.09 | 1.03 (0.99-1.07) |
|  | 23 | MR Egger | 0.075 | 0.054 | 0.18 | 1.08 (0.97-1.20) |
|  | 23 | Simple mode | 0.063 | 0.049 | 0.22 | 1.07 (0.97-1.17) |
|  | 23 | Weighted median | 0.026 | 0.025 | 0.31 | 1.03 (0.98-1.08) |
|  | 23 | Weighted mode | -0.027 | 0.047 | 0.57 | 0.97 (0.89-1.07) |
| Ascorbate | 14 | IVW | -0.042 | 0.050 | 0.41 | 0.96 (0.87-1.06) |
|  | 14 | MR Egger | -0.027 | 0.091 | 0.77 | 0.97 (0.81-1.17) |
|  | 14 | Simple mode | 0.029 | 0.098 | 0.77 | 1.03 (0.85-1.25) |
|  | 14 | Weighted median | -0.019 | 0.066 | 0.77 | 0.98 (0.86-1.12) |
|  | 14 | Weighted mode | -0.035 | 0.056 | 0.54 | 0.97 (0.86-1.08) |

IVW: inverse variance weighted.

**
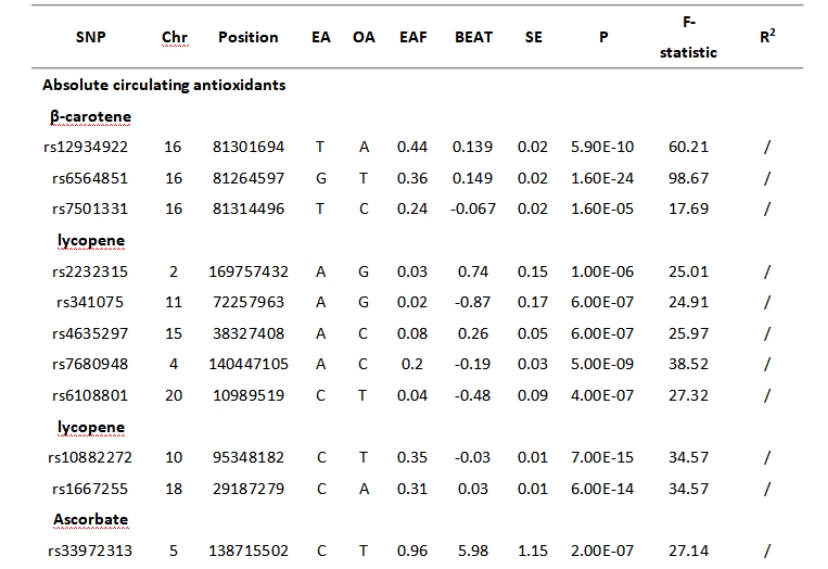
**

**
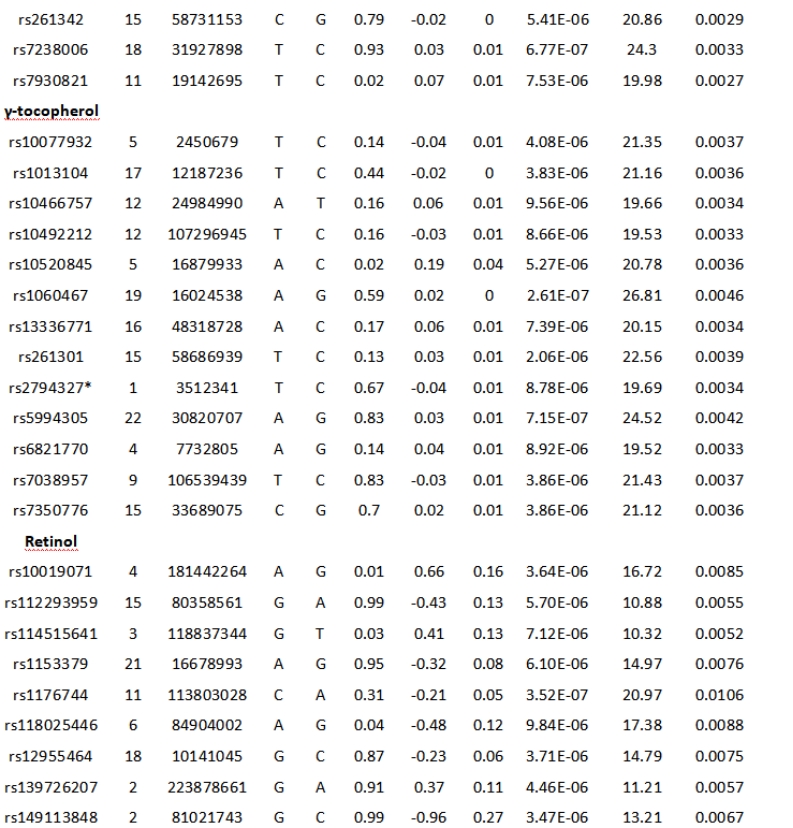

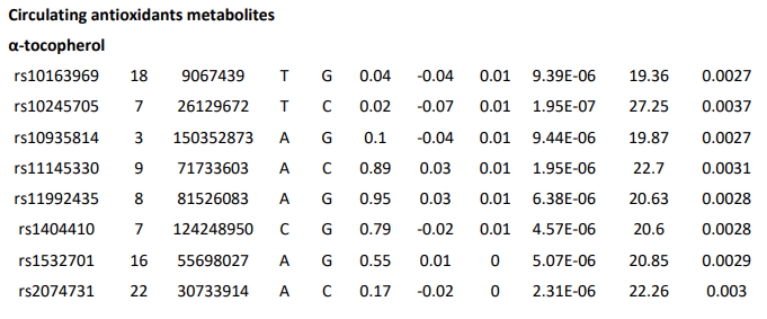
**

**
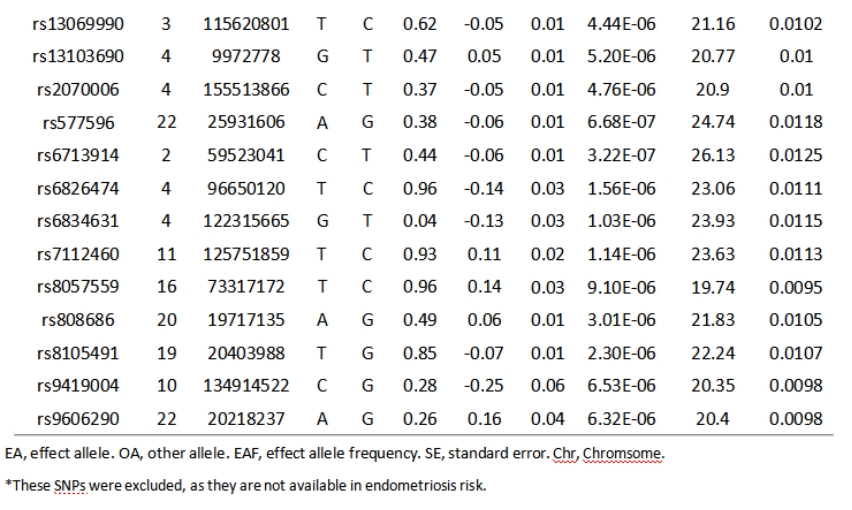

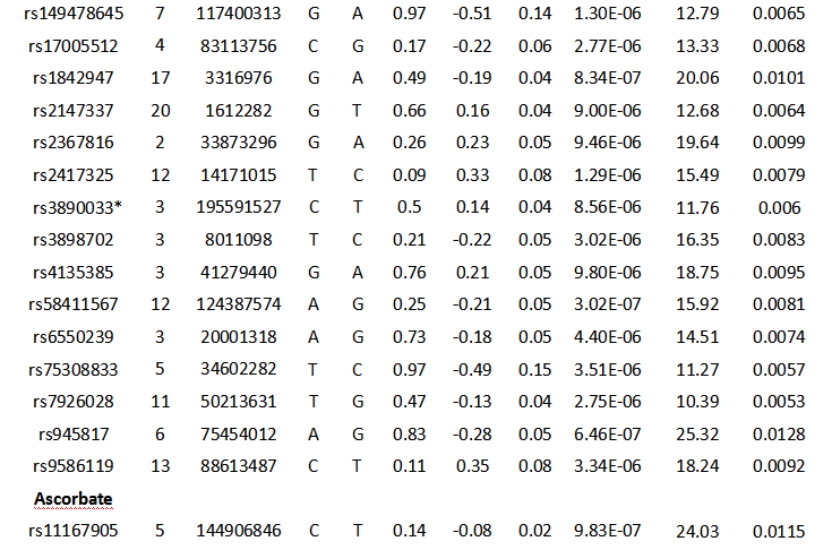
**

**Supplementary Table S3. The results of mendelian randomization between circulating antioxidants and endometriosis**

| **antioxidants** | **nSNP** | **method** | **Beta** | **SE** | **P-value** | **OR (95%CI)** |
| --- | --- | --- | --- | --- | --- | --- |
| β-carotene | 2 | IVW | -0.051 | 0.095 | 0.59 | 0.95 (0.79-1.15) |
| lycopene | 5 | IVW | 0.014 | 0.032 | 0.67 | 1.01 (0.95-1.08) |
|  | 5 | MR Egger | 0.093 | 0.054 | 0.18 | 1.10 (0.99-1.22) |
|  | 5 | Simple mode | -0.040 | 0.060 | 0.54 | 0.96 (0.85-1.08) |
|  | 5 | Weighted median | 0.008 | 0.037 | 0.83 | 1.00 (0.94-1.08) |
|  | 5 | Weighted mode | 0.032 | 0.039 | 0.46 | 1.03 (0.96-1.11) |
| Retinol | 2 | IVW | -0.483 | 0.332 | 0.15 | 0.62 (0.32-1.18) |
| Ascorbate | 1 | Wald ratio | 0.001 | 0.008 | 0.92 | 1.00 (0.99-1.02) |

IVW: inverse variance weighted.

**Supplementary Table S4. The results of mendelian randomization between circulating antioxidants metabolites and endometriosis**

| **antioxidants** | **nSNP** | **method** | **Beta** | **SE** | **P-value** | **OR (95%CI)** |
| --- | --- | --- | --- | --- | --- | --- |
| r-tocopherol | 10 | IVW | 0.043 | 0.122 | 0.72 | 1.04 (0.82-1.33) |
|  | 10 | MR Egger | 0.010 | 0.258 | 0.97 | 1.01 (0.61-1.67) |
|  | 10 | Simple mode | 0.141 | 0.214 | 0.53 | 1.15 (0.76-1.75) |
|  | 10 | Weighted median | 0.087 | 0.157 | 0.58 | 1.09 (0.80-1.49) |
|  | 10 | Weighted mode | 0.119 | 0.19 | 0.55 | 1.13 (0.78-1.64) |
| α-tocopherol | 8 | IVW | -0.092 | 0.238 | 0.70 | 0.91 (0.57-1.46) |
|  | 8 | MR Egger | -0.766 | 0.779 | 0.36 | 0.47 (0.10-2.14) |
|  | 8 | Simple mode | -0.238 | 0.525 | 0.66 | 0.79 (0.28-2.21) |
|  | 8 | Weighted median | -0.186 | 0.328 | 0.57 | 0.83 (0.44-1.58) |
|  | 8 | Weighted mode | -0.352 | 0.483 | 0.49 | 0.70 (0.27-1.81) |
| Retinol | 23 | IVW | 0.031 | 0.018 | 0.09 | 1.03 (0.99-1.07) |
|  | 23 | MR Egger | 0.075 | 0.054 | 0.18 | 1.08 (0.97-1.20) |
|  | 23 | Simple mode | 0.063 | 0.049 | 0.22 | 1.07 (0.97-1.17) |
|  | 23 | Weighted median | 0.026 | 0.025 | 0.31 | 1.03 (0.98-1.08) |
|  | 23 | Weighted mode | -0.027 | 0.047 | 0.57 | 0.97 (0.89-1.07) |
| Ascorbate | 14 | IVW | -0.042 | 0.050 | 0.41 | 0.96 (0.87-1.06) |
|  | 14 | MR Egger | -0.027 | 0.091 | 0.77 | 0.97 (0.81-1.17) |
|  | 14 | Simple mode | 0.029 | 0.098 | 0.77 | 1.03 (0.85-1.25) |
|  | 14 | Weighted median | -0.019 | 0.066 | 0.77 | 0.98 (0.86-1.12) |
|  | 14 | Weighted mode | -0.035 | 0.056 | 0.54 | 0.97 (0.86-1.08) |

IVW: inverse variance weighted.

**
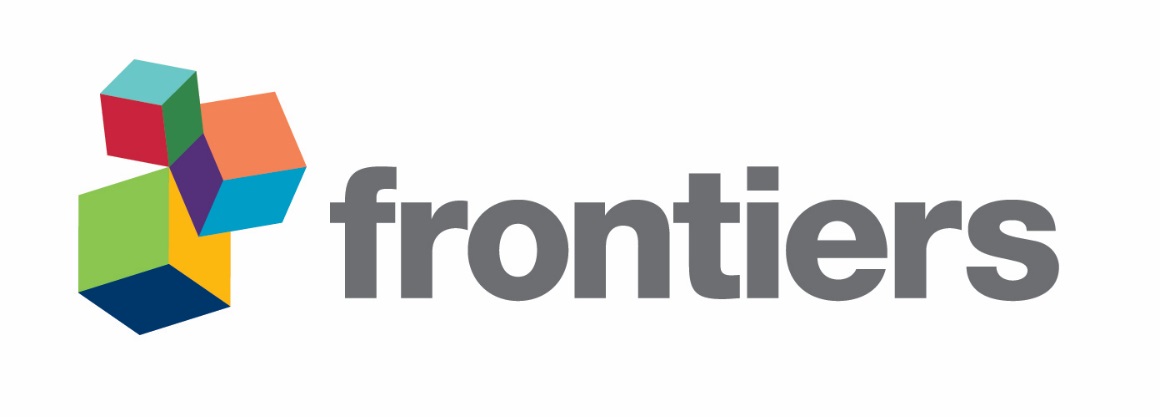
**

**Supplementary Figure 1.** The figure legends are required to have the same font as the main text, 12 point normal Times New Roman, single spaced. Please use a single paragraph for each legend and prepare the figures keeping in mind the PDF layout.
